# Fatty Acid and Phenolic Profiles of Virgin Olive Oils from Local and European Varieties Planted in Lebanon

**DOI:** 10.3390/plants12142681

**Published:** 2023-07-18

**Authors:** Milad El Riachy, Peter Moubarak, Ghenwa Al Hawi, Myriam Geha, Walid Mushantaf, Nathalie Estephan, Wadih Skaff

**Affiliations:** 1 Department of Olive and Olive Oil, Lebanese Agricultural Research Institute, Zahleh P.O. Box 287, Lebanon; moubarak.p@gmail.com (P.M.); ghinwahawi84@hotmail.com (G.A.H.); myriam.geha@hotmail.com (M.G.); 2 Boustan Al Zaytoun, Subsidiary of Green and Pure, Aabra Village, Main Road, Aabra Saida 1600, Lebanon; walid@bustanelzeitoun.com; 3 Department of Chemistry and Biochemistry, Faculty of Arts and Sciences, Holy Spirit University of Kaslik, Jounieh P.O. Box 446, Lebanon; 4 Food Industry and Agriculture Unit, ESIAM, Faculty of Engineering, Saint Joseph University of Beirut, Beirut P.O. Box 17-5208, Lebanon; wadih.skaff@usj.edu.lb

**Keywords:** *Olea europaea* L., virgin olive oil quality, genetic variability, geographical origin, harvesting time, NIR spectroscopy

## Abstract

In Lebanon, olive oil is an integral part of its history and culinary traditions. However, the quality of this product, originating from different growing regions of the country, is rarely addressed. The objectives of this study were to compare the fatty acids and phenolic profiles of virgin olive oils produced from two local and eight European varieties, and to use these profiles as a tool for their characterization. Seventy-six samples were collected from two olive-growing regions of Lebanon and at two harvesting times. Fatty acid composition was determined by gas chromatography with a flame ionization detector, total phenols was determined by spectrophotometry and individual phenols by high performance liquid chromatography with diode array detector. All samples were also analyzed using near infrared spectroscopy. The experimental data were collected in numerical matrices and treated by chemometric methods. The results showed the significant effect of the geographical origin, the olive variety and the harvesting time on the ripening and industrial yield of the olives and on the major fatty acids and phenols of olive oil. Moreover, the used chemometric methods allowed the discrimination of European olive varieties based on their high contents of oleic acid, oleacein and oleocanthal.

## 1. Introduction

The olive tree (*Olea europaea* L.) is an evergreen tree endemic to the Mediterranean region and strongly adapted to its climate. For more than 5000 years, the olive fruit and the virgin olive oil (VOO) are one of the best known and most used products in this region [1]. VOOs extracted from healthy olive fruits preserve the taste, aroma, vitamins and properties of these fruits; and thus, this oil is also characterized with peculiar organoleptic characteristics [2]. Nowadays, VOO is considered as a central feature in the Mediterranean diet and is achieving growing interest as a result of its nutritional, antioxidant and pharmaceutical properties. The key of these properties is its well-balanced fatty acid composition and a plethora of phenolic compounds [3,4]. In fact, VOO is characterized by a high content of monounsaturated fatty acids, mainly oleic acid, that may reach 70 to 85%, as well as lower concentrations of polyunsaturated fatty acids, mainly linoleic and linolenic fatty acids, and of saturated fatty acids, mainly palmitic and stearic fatty acids [5]. This fatty acid profile and a high ω6/ω3 ratio provide the oil with protective effects against cardiovascular diseases, thrombosis, autoimmune and inflammatory disorders and several kinds of cancers [6,7,8]. Besides fatty acids, the phenolic compounds in VOO present powerful antioxidant and anti-inflammatory properties that enables them to slow down the progression of cardiovascular, neurodegenerative and cancer diseases [9]. Moreover, a large part of the unsaponifiable fraction of VOO is composed of squalene and sterols known for their protective effect against several kinds of cancers; in addition to several other minor compounds such as pigments and flavor compounds [10,11]. On the other hand, the fatty acid and phenolic profiles of VOO are the main factors that affect its oxidative stability [12]. It is worth to note that, phenolic compounds strongly contribute to the unique flavor of olive oil [13]. Several factors may affect olive fruit composition and consequently VOO composition. These could be classified as pre-harvest such as geographical origin, variety, irrigation, fertilization and harvesting time; and, as post-harvest such as olive transportation, olive storage and olive processing [14]. The variety and the pedoclimatic conditions of the olive-growing region, including altitude, rainfall, temperature and soil characteristics, are critical variables that affect the evolution of the ripening process and thus the composition of the olive fruits and subsequently of the produced olive oil [15]. In general, a parameter that may indicate the evolution of the ripening process is the fruit skin color. Green olives are rich in phenolic compounds and present fruity and bitter VOOs that have high oxidative stability, but result in a smaller oil yield; however, black fruits present VOOs with a lower content of phenolic compounds and a lower oxidative stability, but a higher oil yield. Turning color fruits, i.e., in the middle of the ripening process, provide the best quality of VOO in terms of aroma, flavor, composition and oxidative stability as well as the best ratio of quality/quantity [16,17].

Nowadays, olive oil quality monitoring through a reliable, cheap and fast technique imposes itself. Several tools have been tested for assessing the authentication and traceability of VOO and for verifying its geographical origin, such as nuclear magnetic resonance (NMR), mass spectrometry (MS) and near infrared spectroscopy (NIRS) coupled with chemometrics [18,19,20]. Near infrared spectroscopy coupled with chemometric methods is thus presented as a rapid, non-destructive, reliable and inexpensive analytical method that can be used in the quality control of olive oil. It can be used to run qualitative and quantitative analysis on olive oil samples [21,22]. For instance, it was used by Cayuela et al. (2009) [23] to classify olive oil samples based on the function of the used processing system and the geographical origin. Additionally, Shaik (2008) [22] used the FT-IR technique to establish prediction models for olive oil quality parameters (fruit moisture, free acidity, etc.) as an alternative to classical chemical methods that require chemical reagents as well as a large amount of time and that generate wastes. Moreover, Valli et al. (2016) [20] applied the near infrared spectroscopy to detect adulteration among olive oil samples.

Lebanon is renowned for its unique tasting olives, and Lebanese VOO is one of the country’s best-known agro-industrial products and is among the most expensive olive oils in the world due to its exceptional quality, color and taste. The olive sector is an important economic and socio-cultural activity in many parts of Lebanon. In 2020, olive trees occupied an area of 74,188 ha with a production volume of about 160,000 tons and an olive oil production of approximately 18,000 tons [24]. The main olive-growing zones in Lebanon are in the north of the country (41%) and the south of the country (36%); with smaller percentages in the Bekaa region (13%) and the Mount Lebanon region (10%) [25]. The traditional olive groves are planted mainly with the variety ‘Baladi’ (which means from my country), representing around 70% of the olive production in the country, followed by the variety ‘Soury’ (originating from the city “Tyre” in South Lebanon, called “Sour” in Arabic), representing around 20% [26]. However, for new plantations, farmers are searching for new olive varieties with improved characteristics. The most well-known imported varieties in Lebanon are the Italian varieties (mainly ‘Frantoio’, ‘Coratina’, ‘Pendolino’ and Leccino’), the Spanish varieties (mainly ‘Arbequina’ and ‘Manzanilla de Sevilla’), the greek varieties (mainly ‘Kalamata’ and ‘Koroneiki’), the Jordanian varieties (mainly ‘Nabali’) and the Moroccan varieties (mainly ‘Picholine Marocaine’). For this reason, the objectives of this study were: (1) to compare the composition of VOOs obtained from some European varieties (‘Bianchera’, ‘Coratina’, ‘Frantoio’, ‘Leccino’, ‘Maurino’, ‘Pendolino’, ‘Sant Agostino’ and ‘Verzola’) to that of the two main indigenous varieties (‘Baladi’ and ‘Soury’) planted in North and South Lebanon and (2) to use the fatty acids and the phenolic profiles as a tool for their characterization.

## 2. Results

A 3-way ANOVA was conducted to check the effects of the three studied factors (geographical origin, harvesting time and variety) on the ripening index, the industrial yield and the total phenolic compounds and subsequently for all the required assumptions.

### 2.1. Effect on the Ripening Process

Detailed ANOVA results concerning the ripening index, calculated based on fruit skin color that may indicate the evolution of the ripening process, are shown in Table 1.

The geographical origin and harvesting time presented, respectively, a high significant effect on the ripening (*p* < 0.01), whereas the variety showed a very high significant effect. It is worth to note that the power for each of the studied factors was very high (≥0.86). The eta-squared value (*η*^2^) of the variety indicates that the observed differences among varieties are explained at 89.23% by this factor.

It is important to highlight the fact that the two-way interactions (geographical origin × harvesting time) and (geographical origin × variety) showed, respectively, a statistical (*p* < 0.05) and very high statistical (*p* < 0.001) significant effect on the ripening, which means that regardless of the olive oil’s origin, the harvesting time and the variety affect the calculated value of the ripening index.

### 2.2. Effect on the Industrial Yield

The detailed analysis related to industrial yield is detailed in Table 2.

Each of the three main factors shows a very high significant effect on the industrial yield (*p* < 0.001). Note that the power for each of the factors was very high (≥0.98). Concerning the eta-squared value ($\eta^{2}$), it shows that the geographical origin, the harvesting time and the variety explain, respectively, 61.25%, 32.26% and 85.27% of the observed differences.

It is important to highlight the fact that the two-way interactions (geographical origin × harvesting time) and (geographical origin × variety) showed, respectively, a statistical (*p* < 0.05) and very high statistical (*p* < 0.001) significant effect on the olive oil’s industrial yield.

### 2.3. Effect on the Total Phenolic Content (TPC)

The analysis of the effect of the studied factors on the TPC is presented in Table 3.

Concerning the TPC, only the variety showed a very high significant effect on the TPC (*p* < 0.001). For this factor, the calculated power is equal to 1 and the eta-squared value (*η*^2^) is equal to 64.19%.

The two-way interactions (geographical origin × variety) was the only one showing a high statistically significant effect (*p* < 0.01) on the TPC.

Regarding the fatty acids and the individual phenols, two MANOVA were conducted to assess the effect of the different studied factors and their interactions on these profiles. After applying the Bonferroni correction for the tests of between-subjects effects, a result is considered as statistically significant for a “*p*” value less than 0.004 (13 fatty acids) and 0.006 (nine individual phenols).

### 2.4. Effect on the Fatty Acids

The results of the multiple analysis of variance (MANOVA) on the fatty acid profile are shown in Table 4.

A very high significant effect of the geographical origin and the variety was observed on the fatty acid profile of the virgin olive oil (*p* < 0.001). It is worth noting that the calculated power was equal to 1, respectively, and the eta squared was very high (88.35% and 96.55%, respectively), indicating that the two mentioned factors explains 88.35% and 96.55% of the observed variability, respectively, of the fatty acid profile among regions and varieties. In addition, the harvesting time showed a significant effect on the fatty acid profile of the virgin olive oil with a power equal to 0.92 and an eta squared of 58.76%. Moreover, the two-way interaction (geographical origin × variety) showed a very high significant effect on the fatty acid profile (Wilks’ Λ = 0.00; F (117;200.345) = 4.46; *p* < 0.001). The observed power and the eta-squared values were equal to 1 and 72.26%, respectively, indicating a high capacity for MANOVA to detect such differences among means and high effect (72.26%) of the two-way interaction on this set of dependent variables (fatty acids).

Since MANOVA shows a statistically significant effect on the fatty acids, the test of between-subjects effects was performed. The results showed a significant effect (*p* < 0.004) of the geographical origin on C14:0 (0.01% in both regions), C16:0 (14.90% in Aabra and 14.04% in Al Abdeh), C16:1 (0.92% in Aabra and 0.74% in Al Abdeh), C18:0 (2.77% in Aabra and 3.08% in Al Abdeh), C18:3 (0.68% in Aabra and 0.80% in Al Abdeh), C20:0 (0.42% in Aabra and 0.46% in Al Abdeh), C20:1 (0.27% in Aabra and 0.31% in Al Abdeh) and C24:0 (0.12% in Aabra and 0.13% in Al Abdeh).

On the other hand, no significant effects of harvesting time were observed on the different fatty acids (*p* > 0.004).

Finally, the variety factor showed a statistically significant effect on all of the fatty acids (*p* < 0.004). The results of the Duncan post hoc tests are detailed in Table 5.

### 2.5. Effect on the Phenolic Compounds

The results of the analysis of the phenolic compounds by MANOVA are described in Table 6.

The geographical origin and the variety showed a very highly significant effect on the phenolic compounds in the virgin olive oil (*p* < 0.001) with a calculated power equal to 1. Both recorded high eta squared results (72.90% and 68.45%, respectively), indicating that the two mentioned factors explains 72.90% and 68.45% of the observed variability, respectively, of the phenolic profile among regions and varieties. Moreover, the harvesting time showed a significant effect on the phenolic compounds in the virgin olive oil. The calculated power was equal to 0.87 and the eta squared to 45.59%. Furthermore, the two-way interaction (geographical origin × variety) showed a very high significant effect on the phenolic profile (Wilks’ Λ = 0.01; F(81;196.382) = 2.76; *p* < 0.001). The MANOVA power was equal to 1 and the calculated eta-squared value was equal to 53.24%.

The test of between-subjects effects was performed for the factors having significant effects on the phenolic profile. Only vanillic acid and apigenin were significantly affected by the geographical origin (*p* < 0.006). Vanillic acid recorded 3.14 mg/Kg in Aabra and 2.95 mg/Kg in Al Abdeh; while apigenin registered 2.65 and 4.11 mg/Kg, respectively, in Aabra and Al Abdeh.

Similarly to the fatty acids, no significant effects of harvesting time were observed on the different phenolic compounds (*p* > 0.006).

As per the variety factor, it showed a statistically significant effect on vanillic acid, *p*-coumaric acid, oleacein, oleocanthal, luteolin and apigenin (*p* < 0.006). The results of the Duncan post hoc tests for these phenolic compounds are described in Table 7.

### 2.6. Principal Component Analysis (PCA) on the NIR Data in the Function of the Olive Varieties

After the acquisition of NIR spectra, the numerical spectral data were gathered in a matrix X of size (224, 2982) with 76 examples and their replicates and 2982 variables addressing the wavenumbers. The preprocessing of the data was executed using standard normal variate (SNV) to correct the baseline drift in the signal. Then, the PCA was applied on the preprocessed data and calculated using two principal components with the aim to explore the samples distribution in the function of the olive varieties.

The olive varieties were divided into two groups: local varieties (Baladi, Soury) and European varieties (Bianchera, Coratina, Frantoio, Leccino, Maurino, Pendolino, Sant Agostino, Verzola).

**Figure 1:**
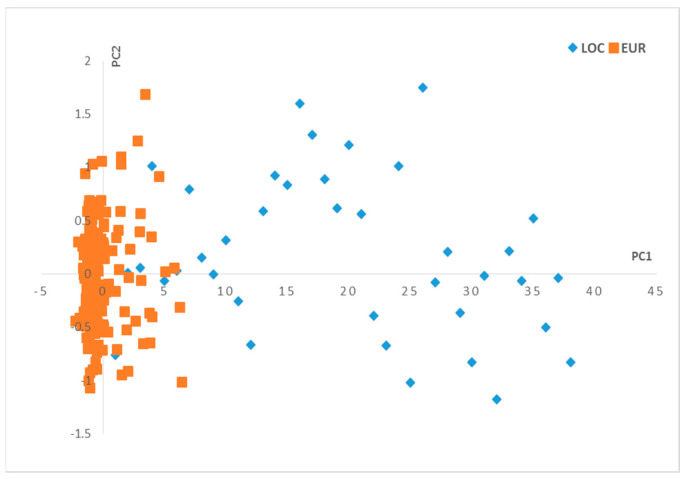
Scores plot for the principal components (PC1–PC2). Local varieties (LOC), European varieties (EUR).

**Figure 2:**
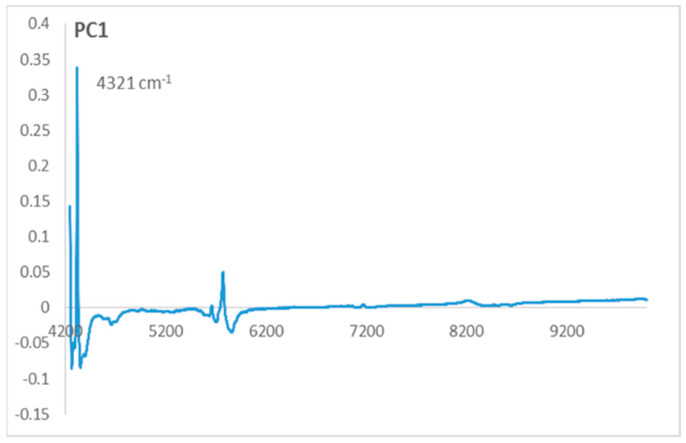
Loadings of the first principal component (PC1).

The PCA application on the NIR data in the function of the olive varieties generates results in terms of scores and loadings, where two principal components, PC1 and PC2, were used for the visualization of the scores (Figure 1) and loadings (Figure 2).

The observations on the scores (Figure 1) show that the two groups of olive varieties are well separated on the PC1 axis: the European varieties are located on the negative side, and the local varieties are more dispersed on the positive side along the PC1 axis.

To understand the separation of the two varieties groups on PC1, we should observe the loadings of PC1 (Figure 2). On the PC1 loadings, we can observe that the only variable that contributes positively on PC1 corresponds to the wavenumber 4321 cm^−1^. This variable characterizes the samples located on the positive side of PC1, which belong to the local olive varieties.

This wavenumber contributing positively on PC1 loadings corresponds to the functional group –CH=CH– which characterizes the local olive varieties group located in the positive side of the PC1 axis. This implicates that these varieties are richer in unsaturated fatty acids than the European varieties.

### 2.7. Factorial Discriminant Analysis (FDA) on Fatty Acid Profiles Generated by Chromatography

**Figure 3:**
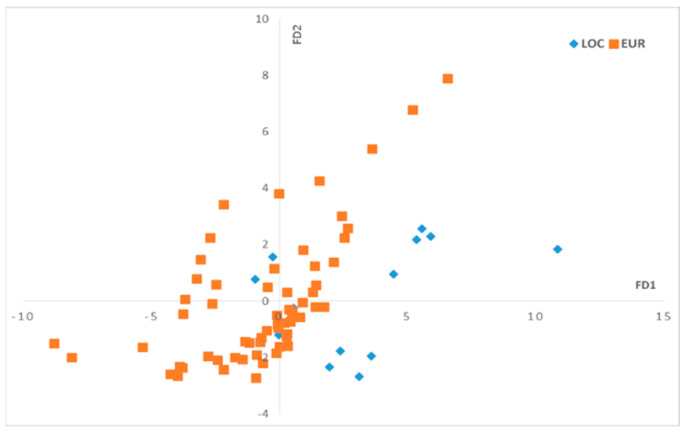
Scores plot of discriminant factors (DF1–DF2) for the fatty acid profiles. Local varieties (LOC), European varieties (EUR).

**Figure 4:**
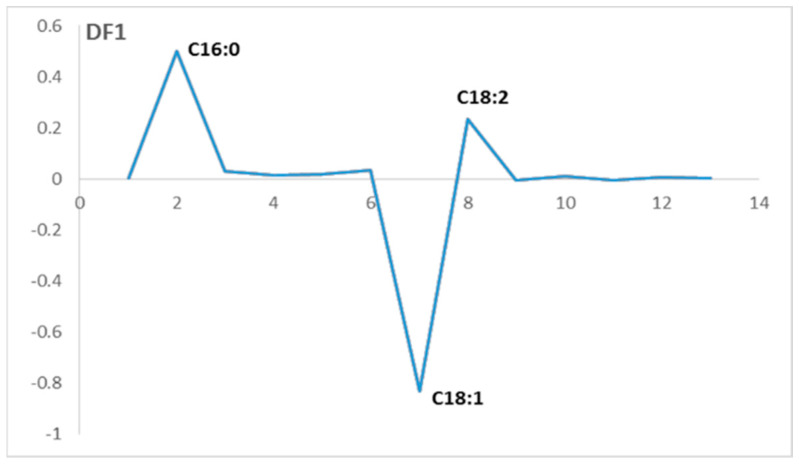
Loadings of discriminant factor (DF1) for the fatty acid profile.

The numerical chromatographic data were collected in a matrix X of size (224, 13) with 76 examples and their replicates and 13 variables corresponding to the identified fatty acid peaks. The FDA was then calculated in the function of the olive varieties using two discriminant factors, DF1 and DF2. Figure 3 displays the scores, while Figure 4 shows the loadings.

On the DF1–DF2 scatter plot (Figure 3), we can observe that the oil samples that correspond to the local olive varieties are mostly located on the positive side of FD1, while the olive oils that correspond to European olive varieties are more dispersed and mostly located on the negative side on the DF1 axis. To understand the separation between these two groups related to olive varieties along the DF1 axis, we should observe the loadings of DF1 (Figure 4).

There is one variable that contributes negatively on the loadings of DF1, which characterizes the group that is placed on the negative side of the DF1 axis. On the other hand, there are two variables that contribute positively on the loadings of DF1, which characterize the samples that are placed on the positive side of the DF1 axis.

The variable that contributes negatively correspond to the fatty acid C18:1, which is the oleic acid. This variable characterizes the olive oils from European olive varieties, mainly located on the negative side of DF1, which means that these varieties have an oleic acid content higher than that observed in the local varieties.

However, the two variables that contribute positively on the DF1 loadings correspond to the fatty acids: C16:0 (palmitic acid) and C18:2 (linoleic acid). These variables characterize the samples mainly located on the positive side of DF1, which correspond to olive oils that come from local olive varieties.

This implicates that these olive varieties contain more palmitic acid and linoleic acid in their fatty acid profiles than that of the European varieties. Table 8 represents the FDA classification map that presents the fitting percentage of the two groups of olive varieties.

As per the FDA classification map, the local samples have a high fitting percentage, two are classified as European samples, which means that 81.8% of the local samples have similarities in their fatty acid composition with this group. The European samples have a high fitting percentage, eight classified as local samples, which means that 87.5% of the European samples have similarities in their fatty acid composition with the other group.

### 2.8. FDA on Phenolic Composition by Chromatography

**Figure 5:**
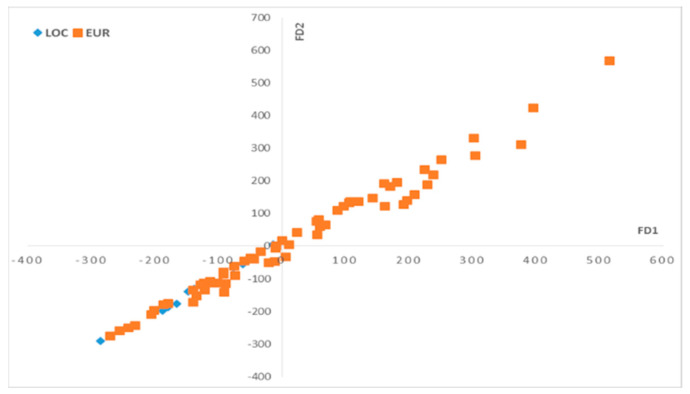
Scores plot of discriminant factors (DF1–DF2 for the phenolic composition. Local varieties (LOC), European varieties (EUR).

**Figure 6:**
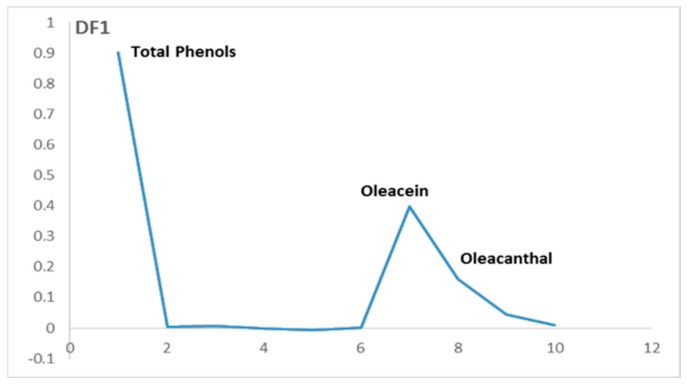
Loadings of the discriminant factor (DF1) for the phenolic composition.

The numerical chromatographic data were gathered in a matrix X of dimensions (224, 10), containing 76 samples and their replicates, and 10 variables representing the identified phenolic compounds peaks. The FDA analysis was conducted based on the olive varieties, utilizing two discriminant factors, DF1 and DF2. The resulting scores and loadings are visualized in Figure 5 and Figure 6, respectively.

On the DF1–DF2 scatter plot (Figure 5), we can observe that the oil samples that correspond to the local olive varieties are mostly located on the negative side of DF1, while the olive oils that correspond to the European olive varieties are located on both sides on the DF1 axis.

To understand the separation between these two groups related to olive varieties along the DF1 axis, we should observe the loadings of DF1 (Figure 6). The three variables that contribute positively on the loadings of DF1 characterize the group that is placed on the positive side of the DF1 axis. On the other hand, there are no variables that contribute negatively on the loadings of DF1.

The variables that contribute positively on DF1 correspond to the total phenols, oleacein and oleocanthal. These variables characterize the samples mainly located on the positive side of FD1, and it is related to some of the European olive varieties. This implicates that these varieties contain more phenols, oleacein and oleocanthal than the others.

Table 9 represents the FDA classification map that displays the fitting percentage of the two groups of olive varieties. This implicates that these olive varieties contain more total phenols, oleacein and oleocanthal in their phenolic profiles than that of the European varieties.

As per the FDA classification map, the local samples has a high fitting percentage, two are classified as European samples, which means that 81.8% of the local samples have similarities in their phenolic composition with this group. The European samples have a high fitting percentage, with 24 classified as local samples, which means that 62.5% of the European samples have similarities in their phenolic composition with the other group.

## 3. Discussion

Before introducing and disseminating any new variety in a country, it is essential to assess its adaptation to the pedoclimatic conditions of the country and compare its performance to that of the local indigenous varieties. For this reason, this study describes the behavior of eight imported and two local olive varieties in two of the main olive-growing regions of Lebanon at two stages of ripening; and characterizes their ripening, industrial yield as well as the fatty acid and phenolic profiles of the olive oils produced from these varieties.

The results of this study highlighted the strong effect of the geographical origin, olive variety and harvesting time on the ripening. These results are consistent with those of Gharbi et al. (2015) [27] who revealed that the ripening varies according to the genetic factor, the cultivation area, the climatic conditions and the harvesting time. Al Abdeh at the sea level (altitude 18 m above sea level) recorded a higher average of ripening index than Aabra (altitude 180 m above sea level). Among the varieties, ‘Maurino’ and ‘Leccino’ ripened earlier than the other imported varieties; however, the local varieties ‘Baladi’ and ‘Soury’ ripened very late in comparison to the others. These results are in partial agreement with those obtained by Rondanini et al., 2011 [28], who found that ‘Leccino’ ripened the earliest among the seventeen studied varieties, followed by ‘Frantoio’ and then ‘Coratina’.

Similarly, the oil industrial yield appears to be strongly affected by the geographical origin, the olive variety and the harvesting time. This is in complete agreement with the results obtained by Rondanini et al. (2014) [29] who proved that the oil industrial yield of the olive fruits depends on the growing area and environmental conditions, the variety and the ripening stage. Although the ripening was earlier in Al Abdeh, the oil industrial yield recorded in Aabra was significantly higher, thus confirming the strong effect of the geographical origin on this parameter. As per the harvesting time, the fruits harvested in the intermediate harvest period recorded a higher oil industrial yield than those harvested earlier, thereby confirming the fact that the oil industrial yield is progressively increasing until the ripening index 3 is reached, and then it either remains constant or slightly decreases [30]. Although ‘Bianchera’ recorded the highest oil industrial yield (24.43%) as a mean value, ‘Leccino’, ‘Maurino’ and ‘Verzola’ grown in Aabra recorded 25.07%, 23.64% and 23.36% during intermediate harvest, respectively. Therefore, the two Italian varieties ‘Leccino’ and ‘Maurino’ are as productive in Lebanon as in Italy, where their oil yields are, respectively, 18 to 21% and 22% [31].

On the other hand, the TPC was only affected by the variety and by the interaction (geographical origin × variety). This means that regardless of the geographical origin, the variety has a highly significant effect on this parameter. These results are in complete agreement with those obtained by El Riachy el al. (2012) [17] who showed that the genotype variance is the main contributor to the variance in comparison to the fruit ripening. ‘Bianchera’, ‘Coratina’, ‘Maurino’ and ‘Pendolino’ recorded a higher TPC (>400 mg GAE/Kg of oil) than the other studied varieties. As shown in Table S1, these results are in agreement with those obtained by Ripa et al. (2008) [32] and Tura et al. (2007) [33]. However, these results are in partial agreement with those obtained by Rotondi et al. (2010) [34] who reported a high TPC for ‘Frantoio’ samples obtained from four Italian regions (Marche, Lazio, Toscana and Umbria) during four consecutive years.

As per the fatty acid composition, the results showed a very high significant effect of the interaction geographical origin × olive variety and of each factor alone on the fatty acid profile of olive oil, along with a significant effect on the harvesting time. These results are consistent with those obtained by Piravi-Vanak et al. (2012) [35] who proved that the fatty acid composition of olive oil is strongly influenced by the variety, the ripening process and the geographical origin, especially the latitude and the climatic conditions. All the obtained percentages of fatty acids fall within the norms established by the IOC for virgin olive oils. On the other hand, and as described by Ollivier et al. (2003) [36], an olive variety is considered to have high C18:1 if the percentage is above 65%. In this study, all the varieties studied showed high C18:1, except for the ‘Baladi’ variety grown in Aabra and harvested on the intermediate harvest date, and the ‘Soury’ variety grown in Al Abdeh and harvested in the early harvest date. As C18:1 is recognized for its high nutritional value and oxidative stability [37,38], it is therefore very important to harvest the ‘Baladi’ variety earlier to obtain virgin olive oils with high C18:1 percentage.

Among the major fatty acids, only C16:0 and C18:0 showed a significant difference between the two studied regions. According to Beltrán et al. (2004) [39], the air temperature during oil biosynthesis could affect the amount of polyunsaturated fatty acids (linoleic and linolenic fatty acids) through the regulation of desaturase enzymes activities; and according to Mailer et al. (2010) [40], a higher content of C18:1 was observed in cooler regions (high altitudes). However, this was not observed in this study because of the small difference in latitude between the two considered regions.

On the other hand, the test of between-subjects effects showed that the variety has a significant effect on all the studied fatty acids. However, the discussion will be limited to C18:1 as it is the major fatty acid in virgin olive oil, as well as due to its impact on human health and on olive oil quality. A comparison between the results obtained in the current study and the data found in the literature on the C18:1 percentages is presented in Table S2. As it can be seen, these results are in agreement with those obtained by the following authors: Dabbou et al. (2015) [41] in Tunisia for ‘Coratina’; Mailer et al. (2010) [40] in Australia and Aguilera et al. (2005) [42] in Andalusia (Spain) for ‘Frantoio’; El Riachy et al. (2019) [30] in Lebanon for ‘Baladi’; and Chehade et al. (2012) [43] in Lebanon for ‘Soury’. However, the results reported in the present study are higher than those obtained by Rondanini et al. (2011) [28] for ‘Coratina’, ‘Frantoio’ and ‘Leccino’ planted in Argentina, but are lower than those recorded by Rotondi et al. (2010) [34] for the same varieties planted in Italy. All these results reveal the huge differences in the behavior of the olive varieties in different cultivation zones; in this case, the fatty acid profile. Furthermore, the results stress on the importance of the introduction of new olive varieties to Lebanon, with high oleic acid percentages due to the importance of this fatty acid for human health and for the oxidative stability of the virgin olive oil (García-González et al., 2008) [44].

Regarding the individual phenolic compounds of the virgin olive oil, the results showed a very highly significant effect of the interaction geographical origin × olive variety and of each of these two factors alone on these compounds. In fact, the qualitative and quantitative phenolic profiles of olive oil were described to be strongly affected by the olive variety and by the environmental conditions related to the areas where the olive trees are planted, in particular the climate and the soil [26,45,46,47]. In parallel, the harvesting time appeared to significantly affect the phenolic profiles, similarly to what was described by El Riachy et al. (2012) [17].

As per the test of between-subjects effects, it shows that the harvesting time did not significantly affect the phenolic profiles, mainly due to the reduced interval of time between the two harvests (two weeks). However, the geographical origin only significantly affected the vanillic acid and the apigenin contents. As expected, the variety significantly affected the majority of the phenolic compounds, namely the six following individual phenols: vanillic acid, *p*-coumaric acid, oleacein, oleocanthal, luteolin and apigenin. Among these, the secoiridoid derivatives oleacein (3,4-DHPEA-EDA) and oleocanthal (p-HPEA-EDA) are those with the higher contents and with higher beneficial effects on human health [48,49], and thus, they are compared to previous studies in Table S3. As observed in the latter, the obtained results are in coherence with the data registered by Ragusa et al. (2017) [50] for ‘Frantoio’. However, they are much lower than those recorded by the same authors and by González et al. (2010) [51] for ‘Leccino’. As per ‘Coratina’, the current results are much lower than those reported by Dabbou et al. (2015) [41] and by Servili et al. (2011) [52] for both oleacein and oleocanthal, while being much higher than those obtained by Ben Hassine et al. (2022) [53] (Table S3). This variability could be related to the differences in fruit ripening, pedo-climatic conditions, agricultural practices, etc. Regarding ‘Baladi’ and ‘Soury’, the oleacein and oleocanthal contents were lower than those recorded for some of the introduced varieties. Similarly, the recorded values for ‘Baladi’ were lower than those recorded by El Riachy et al. (2018) [26] for two clones of ‘Baladi’. It is worth to mention here that the contents of oleacein and oleocanthal recorded for ‘Soury’ were close to those obtained for the clone ‘Baladi 1’ in El Riachy et al. (2018) [26] (Table S3).

On the other hand, the PCA analysis performed on NIR spectral data revealed a distinctive separation between two groups of olive varieties, local and European, based on their levels of unsaturated fatty acids. Additionally, the FDA analysis conducted on chromatographic data for fatty acid and phenolic profiles also demonstrated a clear differentiation between the two groups. Specifically, the European olive varieties exhibited a higher concentration of oleic acid, oleacein and oleocanthal, while local varieties were characterized by a greater presence of palmitic and linoleic acids and other phenols.

## 4. Materials and Methods

### 4.1. Experimental Sites and Sampling

This research work was carried out in two of the main olive-growing regions of Lebanon, namely Al Abdeh in the North and Aabra in the South of the country. Al Abdeh is a village of Akkar Governorate, located at 18 m above sea level, 34°31′0″ N and 35°58′0″ E. This region is characterized by a typical Mediterranean climate and a dry summer from June to September. The average annual rainfall is 870 mm. The soil is clay type with 60% clay, 25% sand and 15% silt. Since 1970, an international collection of olive trees comprising 72 local, Arab and European varieties was established in the Al Abdeh station of the Lebanese Agricultural Research Institute (LARI). On the other hand, Aabra is a village located at the east of Sidon city in the South Governorate. Aabra is located at 180 m above sea level, 33°34′0″ N and 35°24′17″ E. Similarly, to Al Abdeh, Aabra is also characterized by a typical Mediterranean climate with a dry summer from June to September and an average annual rainfall of 778 mm. The soil is of calcareous brown type. In 2011, Walid Mushantaf, the owner of ‘Bustan El Zeitoun’ company for olive oil production, preserved a hill by planting 6000 olive trees of a dozen local and European varieties over an area of 25 ha. Two local olive varieties, ‘Baladi’ and ‘Soury’, and eight European varieties, ‘Bianchera’, ‘Coratina’, ‘Frantoio’, ‘Leccino’, ‘Maurino’, ‘Pendolino’, ‘Sant Agostino’ and ‘Verzola’, were studied. These European varieties were selected because they are common to the two experimental sites. From each variety, two trees were selected in Al Abdeh and two in Aabra. Then, around one kg of olive fruits were harvested at two different times: the first at the beginning of the harvesting season (early harvest, at mid-September) and the second after two weeks (late harvest, at the beginning of October). A total of 76 samples were collected, because in Al Abdeh, only one tree of the ‘Soury’ variety had fruits and only for one single harvest (early harvest), and one tree of the ‘Sant Agostino’ variety had fruits only for the early harvest.

### 4.2. Ripening Index Calculation

The ripening index calculated based on fruit skin color was used as this is a parameter that may indicate the evolution of the ripening process. The ripening index of each sample was determined as described by Uceda and Hermoso (1998) [54] but with slight modifications according to El Riachy et al. (2012) [17]. In brief, 30 olive fruits of each sample were classified, according to the color of their skin, into 5 groups:

- Group 0: fruits with green skin;
- Group 1: fruits with yellowish green skin;
- Group 2: fruits in turning color;
- Group 3: fruits with completely colored skin;
- Group 4: fruits with black skin.

Then, the ripening index was calculated using the following formula:

RI = ([(0*n0) + (1*n1) + (2*n2) + (3*n3) + (4*n4)])/30(1)

where, n0, n1, n2, n3 and n4 are the number of fruits in the corresponding group.

### 4.3. Virgin Olive Oil Extraction and Industrial Yield Calculation

VOO was extracted within 24 h of the olive harvest using the Abencor system (MC2 Ingeniería y Sistemas, Seville, Spain), simulating commercial VOO continuous extraction systems. The olives were crushed with a small hammer mill, equipped with a 4 mm sieve. Then, the paste obtained was mixed and homogenized for 30 min at 25–27 °C using a thermo-mixer and finally centrifuged for 2 min to separate the VOO. The extracted VOOs were stored at −20 °C in the dark until their analysis. Industrial yield was calculated according to the formula proposed by Martínez-Suarez et al. (1975) [55]:

IY = (V*0.915*100)/M (2)

where, V is the volume of obtained oil and M is the mass of used olive paste.

### 4.4. Fatty Acid Composition

The fatty acid methyl esters (FAMEs) were prepared according to the reference method of cold transmethylation [56]. An amount of 0.1 g of a VOO sample was manually mixed with 2 mL of n-hexane for 2 s, then 0.2 mL of the methanolic potassium hydroxide solution (2N) was added. The sample was vortexed for 1 min (1400 rpm) and then allowed to stand for 5 min. A volume of 975 μL of the upper phase containing the FAMEs was transferred into 1.5 mL vials with 25 μL of external standard (nonadecanoate methyl ester, 1000 ppm, analytical standard, Sigma-Aldrich Co., St. Louis, MO, USA). FAME separation was performed using a Shimadzu gas chromatograph (GC-2010 Plus, Shimadzu Corporation, Tokyo, Japan) equipped with a flame ionization detector (FID, 280 °C). A fused silica capillary column (DB-wax, Agilent Technologies, Wilmington, DE) with 30 m length, 0.25 mm internal diameter and 0.25 μm of film thickness was used. The carrier gas was nitrogen gas N50 with a flow of 1.69 mL/min. As per the injector, the temperature was fixed at 250 °C with a split ratio of 1:50. A gradient oven temperature program was adopted with initial temperature set at 165 °C for 15 min, then temperature increased from 165 °C to 200 °C at a rate of 5 °C/min, and kept at 200 °C for 2 min, then raised from 200 °C to 240 °C at a rate of 5 °C/min, and finally held at 240 °C for 5 min. The identification of the different fatty acids was carried out by comparing their retention times with those of commercial standards. The quantitation of each compound is expressed in percentage based on the area under the peak of the corresponding compound and the percentage it represents of all compounds.

### 4.5. Phenolic Compounds

Before analysis, samples were thawed at room temperature in the dark. During this time, an internal standard solution was prepared by dissolving 15 mg of syringic acid in 10 mL of 60:40 (*v/v*) methanol/water. Then, 1 mL of this solution was diluted in a 25 mL volumetric flask with 60:40 (*v/v*) methanol/water. Phenolic compounds from olive oil were extracted using the procedure described by Montedoro et al. (1992) [57] with some modifications. A quantity of 3 g of olive oil was manually stirred with 2 mL of n-hexane for 15 s. Then, a volume of 1.75 mL of the 60:40 (*v/v*) methanol/water mixture was added, as well as 0.25 mL of the internal standard solution, and then the whole solution was stirred for 2 min to perform the first extraction. For the second extraction, 2 mL of the 60:40 (*v/v*) methanol/water mixture was added and stirred for 2 min. The extracts from both extractions were combined and placed in the dark at −20 °C for subsequent determinations.

#### 4.5.1. Determination of Total Phenols

The total phenolic content in the VOO extracts was determined according to the spectrophotometric method of Folin–Ciocalteu (F-C) [58]. Briefly, 20 μL of the sample was stirred with 1.58 mL of water, 0.3 mL of the 20% (*w/v*) Na2CO3 aqueous solution and 0.1 mL of the F-C reagent, then heated in an oven at 50 °C for 5 min in order to accelerate the reaction. Then, the resulting solution was allowed to stand for 30 min. The reaction product was analyzed spectrophotometrically at 765 nm using a Jenway UV–visible spectrophotometer.

#### 4.5.2. Determination of Individual Phenols

The phenolic extract produced was also used to determine the following 9 individual phenolic compounds: hydroxytyrosol, tyrosol, vanillic acid, o-coumaric acid, p-coumaric acid, oleacein, oleocanthal, luteolin and apigenin. The extracted phenolic fraction was injected into a Shimadzu high performance liquid chromatograph (HPLC, Shimadzu Corporation, Tokyo, Japan) equipped with an automatic injector, a Microsorb-MV 100 C18-type column (250 × 4.6 id mm, particle size of 5 μ) maintained at 22 °C and a diode array UV detector (DAD, using 280 nm as quantification wavelength). A volume of 20 μL of the phenolic extract was injected. While the mobile phase A was 0.2% of *o*-phosphoric acid, the mobile phase B was a mixture methanol/acetonitrile (50:50, *v/v*). The flow rate was fixed at 1.0 mL/min. The initial concentrations were 96% of A and 4% of B and the gradient was changed as follows: the concentration of B was raised to 50% in 40 min, increased to 60% in 5 min, then to 100% in 15 min, and kept constant for 10 min. Initial conditions were reached in 7 min. Identification of individual phenols in olive oil was performed at 280 nm, based on their retention times compared to those of commercial standards.

### 4.6. Fourier Transform near Infrared Spectrometry (FT-NIR)

#### 4.6.1. Spectra Acquisition

The “Antaris FT-NIR Analyzer” (Thermo Fisher scientific, Waltham, MA, USA) fitted with an InGaAs/germanium detector was the spectrophotometer used for spectra collection. The instrument resolution was fitted at 4 cm^−1^ resolution. For each of the 76 olive oil samples collected, spectra were acquired in triplicates by averaging 50 scans·s^−1^ over the wavenumber range of 3800–12,000 cm^−1^. A background spectra were obtained before each spectrum acquisition.

#### 4.6.2. Pre-Treatments Applied to Spectra

To ensure spectrum data reliability, different pre-treatments and their combinations were tested. The retained one was the standard normal variate (SNV). It was used to correct the baseline drift in the signal.

### 4.7. Statistics and Chemometrics Analysis

#### 4.7.1. Classic Statistical Analysis

Classic statistical analysis of data was performed using IBM SPSS Statistical software (version 25.0). The three-way analysis of variance (ANOVA) was performed to determine the effects of the three independent variables (geographical origin, olive variety and harvesting time) on each of the dependent variables (ripening index, industrial yield and total phenolic compounds). In addition, multivariate analysis of variance (MANOVA) was performed to determine the effects of the three independent variables (geographical origin, olive variety and harvesting time) on the two sets of dependent variables (the 13 fatty acids and the 9 individual phenolic compounds) after checking for all the required variables. Mean comparisons were performed using Duncan’s post hoc test.

#### 4.7.2. Chemometric Analysis

Data preprocessing—standard normal variate (SNV): The method consists of calibrating each spectrum by subtracting the average value and dividing by the standard deviation of all the spectra. This method is useful in reducing the effects of light scattering, while significantly reducing the spectral intensity of certain wavelengths and normalizing them [59,60].

Principal component analysis (PCA): It is a multivariate exploratory method that consists of reducing dimensionality by creating a new set of uncorrelated variables ordered by the amount of variance they explain, allowing for simplified analysis and visualization [61,62]. In this study, PCA was used to assess the relationship between the olive oil fatty acid and phenolic profiles with regard to the studied parameters (geographical origin, variety and harvesting time).

Factorial discriminant analysis (FDA): It is a statistical method used for classification tasks that combines multiple dependent variables to distinguish and classify observations into distinct groups by seeking discriminant axes orthogonal to each other and maximizing the between variance while minimizing the within variance [63].

The data preprocessing and analyses were conducted using the chemometrics software tool CATS 97 (Chemometrics Analysis ToolS).

## 5. Conclusions

In conclusion, the main objective of this study was to compare the composition of VOOs obtained from some European varieties (‘Bianchera’, ‘Coratina’, ‘Frantoio’, ‘Leccino’, ‘Maurino’, ‘Pendolino’, ‘Sant Agostino’ and ‘Verzola’) to that of the two main indigenous varieties of Lebanon (‘Baladi’ and ‘Soury’). The results showed that many of the introduced varieties may produce oils with improved characteristics than the local varieties ‘Baladi’ and ‘Soury’ in terms of oil industrial yield, total phenolic content, C18:1, oleacein and oleocanthal. The study also aimed to use the fatty acid and the phenolic profiles as a tool for the varietal characterization. The NIR tool and the chemometric methods used revealed a distinctive separation between the two groups of olive varieties, local and European ones, based on their fatty acid and phenolic profiles. Finally, these imported varieties could be useful for the implementation of any olive-breeding program in Lebanon for the improvement of the traditional local varieties due to some of their cited relevant characteristics.

## Figures and Tables

**Table 1:** Effect of the geographical origin, harvesting time and variety on the ripening index.

Variables		Ripening Index	F	p	Power	$\eta^{2}$ (%)
Geographical origin	Aabra	1.85 ± 1.10	9.88	0.003 **	0.86	21.08
	Al Abdeh	2.34 ± 1.28				
Harvesting time	Early	1.88 ± 1.21	10.86	0.002 **	0.89	22.69
	Intermediate	2.30 ± 1.19				
Variety	Baladi	0.58 ± 0.64	34.06	0.000 ***	1.00	89.23
	Bianchera	1.10 ± 0.55				
	Coratina	2.58 ± 0.93				
	Frantoio	1.18 ± 0.39				
	Leccino	3.29 ± 0.55				
	Maurino	3.29 ± 0.58				
	Pendolino	3.03 ± 0.74				
	Sant Agostino	2.01 ± 1.14				
	Soury	0.65 ± 0.37				
	Verzaula	2.58 ± 0.78				

Abbreviations in the table indicate: standard deviation (SD), ANOVA statistic (F), probability value (*p*), eta squared ($\eta^{2}$). **: *p* < 0.01; ***: *p* < 0.001.

**Table 2:** Effect of the geographical origin, harvesting time and variety on the industrial yield.

Variables		Industrial Yield (%)	F	p	Power	$\eta^{2}$ (%)
Geographical origin	Aabra	18.20 ± 5.11	58.49	0.000 ***	1.00	61.25
	Al Abdeh	14.76 ± 4.87				
Harvesting time	Early	15.27 ± 4.84	17.62	0.000 ***	0.98	32.26
	Intermediate	17.94 ± 5.40				
Variety	Baladi	16.73 ± 2.62	23.79	0.000 ***	1.00	85.27
	Bianchera	24.43 ± 2.00				
	Coratina	11.78 ± 3.66				
	Frantoio	15.63 ± 3.26				
	Leccino	19.00 ± 4.79				
	Maurino	16.84 ± 4.41				
	Pendolino	14.83 ± 6.23				
	Sant Agostino	16.54 ± 2.89				
	Soury	8.41 ± 1.73				
	Verzaula	18.46 ± 3.39				

Abbreviations in the table indicate: standard deviation (SD), ANOVA statistic (F), probability value (*p*), eta squared ($\eta^{2}$). ***: *p* < 0.001.

**Table 3:** Effect of the geographical origin, harvesting time and variety on the TPC.

Variables		TPC (mg GAE/kg of Oil)	F	p	Power	$\eta^{2}$ (%)
Geographical origin	Aabra	359.91 ± 174.50	1.00	0.324	0.16	2.62
	Al Abdeh	419.50 ± 185.93				
Harvesting time	Early	377.62 ± 164.27	0.05	0.829	0.06	0.13
	Intermediate	399.23 ± 199.34				
Variety	Baladi	336.86 ± 60.38	7.37	0.000 ***	1.00	64.19
	Bianchera	586.64 ± 220.16				
	Coratina	574.06 ± 193.67				
	Frantoio	295.01 ± 73.04				
	Leccino	292.53 ± 182.04				
	Maurino	446.64 ± 101.15				
	Pendolino	433.34 ± 159.92				
	Sant Agostino	233.60 ± 49.64				
	Soury	204.42 ± 86.36				
	Verzaula	390.07 ± 140.31				

Abbreviations in the table indicate: standard deviation (SD), gallic acid equivalent (GAE), ANOVA statistic (F), probability value (*p*), eta squared ($\eta^{2}$). ***: *p* < 0.001.

**Table 4:** MANOVA analysis of the effect on the fatty acid profile.

Variables	Wilks’ Λ	F	p	Power	$\eta^{2}$ (%)
Geographical origin	0.12	14.59	0.000 ***	1.00	88.35
Harvesting time	0.41	2.74	0.015 *	0.92	58.76
Variety	0.00	47.93	0.000 ***	1.00	96.55

Abbreviations in the table indicate: Wilks’ lambda (Wilks’ Λ), ANOVA statistic (F), probability value (*p*), eta squared ($\eta^{2}$). *: *p* < 0.05; ***: *p* < 0.001.

**Table 5:** Fatty acid composition (%) and the sums of saturated (SFA), monounsaturated (MUFA) and polyunsaturated fatty acids (PUFA) (%) for each of the studied varieties.

Variety	C14:0	C16:0	C16:1	C17:0	C17:1	C18:0	C18:1	C18:2	C18:3	C20:0	C20:1	C22:0	C24:0	SFA	MUFA	PUFA
Baladi	0.01 ^c^	14.97 ^b^	0.73 ^d^	0.15 ^a^	0.19 ^a^	3.51 ^c^	67.46 ^ef^	11.15 ^b^	0.68 ^de^	0.53 ^b^	0.29 ^c^	0.16 ^a^	0.09 ^a^	19.41 ^b^	68.66 ^de^	11.84 ^b^
Bianchera	0.01 ^b^	14.11 ^c^	0.85 ^c^	0.04 ^b^	0.05 ^b^	4.42 ^a^	71.12 ^bc^	7.67 ^e^	0.54 ^f^	0.61 ^a^	0.25 ^de^	0.16 ^a^	0.09 ^a^	19.44 ^b^	72.27 ^abc^	8.22 ^c^
Coratina	0.01 ^e^	11.89 ^d^	0.37 ^f^	0.04 ^bc^	0.04 ^bcd^	4.00 ^b^	73.88 ^a^	7.58 ^e^	0.89 ^b^	0.60 ^a^	0.40 ^a^	0.15 ^ab^	0.08 ^ab^	16.77 ^e^	74.69 ^a^	8.46 ^c^
Frantoio	0.01 ^e^	15.35 ^b^	1.10 ^a^	0.03 ^cd^	0.05 ^b^	2.36 ^ef^	70.54 ^bc^	8.99 ^d^	0.65 ^e^	0.39 ^e^	0.26 ^de^	0.12 ^c^	0.07 ^b^	18.32 ^cd^	71.96 ^abc^	9.64 ^bc^
Leccino	0.01 ^d^	14.84 ^bc^	0.89 ^bc^	0.02 ^d^	0.04 ^d^	2.41 ^ef^	70.13 ^c^	10.24 ^c^	0.64 ^e^	0.32 ^f^	0.25 ^de^	0.09 ^d^	0.04 ^d^	17.74 ^cde^	71.31 ^bcd^	10.88 ^bc^
Maurino	0.01 ^de^	14.90 ^b^	0.98 ^b^	0.02 ^d^	0.04 ^d^	2.57 ^e^	68.67 ^d^	11.22 ^b^	0.80 ^bc^	0.34 ^f^	0.25 ^de^	0.09 ^d^	0.04 ^d^	17.97 ^cd^	69.93 ^bcde^	12.03 ^b^
Pendolino	0.01 ^de^	14.62 ^bc^	0.63 ^e^	0.02 ^d^	0.04 ^cd^	2.18 ^fg^	71.54 ^b^	8.99 ^d^	1.01 ^a^	0.35 ^f^	0.33 ^b^	0.12 ^c^	0.06 ^c^	17.37 ^de^	72.54 ^ab^	10.00 ^bc^
Sant Agostino	0.01 ^c^	12.57 ^d^	0.87 ^c^	0.03 ^cd^	0.05 ^bc^	2.01 ^g^	67.98 ^de^	14.69 ^a^	0.77 ^cd^	0.40 ^de^	0.33 ^b^	0.13 ^bc^	0.07 ^b^	15.23 ^f^	69.23 ^cde^	15.46 ^a^
Soury	0.02 ^e^	17.76 ^a^	1.16 ^a^	0.04 ^bc^	0.05 ^bc^	2.57 ^e^	66.65 ^f^	10.11 ^c^	0.73 ^cde^	0.42 ^cd^	0.23 ^e^	0.12 ^c^	0.07 ^b^	20.99 ^a^	68.10 ^e^	10.85 ^bc^
Verzaula	0.01 ^de^	14.90 ^b^	0.89 ^bc^	0.03 ^bcd^	0.05 ^bc^	2.89 ^d^	70.12 ^c^	9.42 ^cd^	0.67 ^de^	0.44 ^c^	0.27 ^cd^	0.14 ^bc^	0.07 ^b^	18.48 ^bc^	71.32 ^bcd^	10.09 ^bc^

Different letters in the same column indicate significant differences.

**Table 6:** MANOVA analysis of the effect on the phenolic compounds.

Variables	Wilks’ Λ	F	p	Power	$\eta^{2}$ (%)
Geographical origin	0.27	8.67	0.000 ***	1.00	72.90
Harvesting time	0.54	2.70	0.02 *	0.87	45.59
Variety	0.00	5.26	0.000 ***	1.00	68.45

Abbreviations in the table indicate: Wilks’ lambda (Wilks’ Λ), ANOVA statistic (F), probability value (*p*), eta squared ($\eta^{2}$). *: *p* < 0.05; ***: *p* < 0.001.

**Table 7:** Phenolic composition (mg/Kg) for each of the studied varieties.

Variety	Vanillic Acid	*p*-Coumaric Acid	Oleacein	Oleocanthal	Luteolin	Apigenin
Baladi	3.31 ^ab^*	1.40 ^a^	16.24 ^c^	26.36 ^c^	4.14 ^b^	2.74 ^de^
Bianchera	2.94 ^cde^	0.12 ^c^	49.19 ^bc^	50.62 ^bc^	11.64 ^a^	4.79 ^a^
Coratina	2.85 ^de^	0.00 ^c^	109.96 ^ab^	70.26 ^b^	11.99 ^a^	3.89 ^bc^
Frantoio	3.21 ^abc^	0.00 ^c^	12.52 ^c^	21.16 ^c^	4.77 ^b^	2.22 ^e^
Leccino	3.40 ^a^	0.06 ^c^	140.27 ^a^	120.03 ^a^	10.45 ^a^	3.06 ^de^
Maurino	3.12 ^bcd^	0.14 ^c^	103.84 ^ab^	52.25 ^bc^	12.58 ^a^	2.33 ^e^
Pendolino	2.81 ^e^	0.00 ^c^	155.20 ^a^	116.59 ^a^	13.50 ^a^	3.35 ^cd^
Sant Agostino	3.02 ^cde^	0.95 ^b^	63.10 ^bc^	144.30 ^a^	4.10 ^b^	4.57 ^ab^
Soury	3.09 ^bcde^	0.30 ^c^	25.05 ^c^	73.02 ^b^	3.08 ^b^	2.33 ^e^
Verzaula	2.81 ^e^	0.00 ^c^	14.36 ^c^	19.28 ^c^	9.36 ^a^	3.90 ^bc^

*: Different letters in the same column indicate significant differences.

**Table 8:** FDA classification map for the fatty acid profiles.

	LOC	EUR	% Fit
LOC	9	2	81.8
EUR	8	56	87.5

Abbreviations in the table indicate: local varieties (LOC), European varieties (EUR).

**Table 9:** FDA classification map for the phenolic composition.

	LOC	EUR	% Fit
LOC	9	2	81.8
EUR	24	40	62.5

Abbreviations in the table indicate: local varieties (LOC), European varieties (EUR).

## Data Availability

The database is available by contacting the corresponding authors (Milad El Riachy: mriachy@lari.gov.lb; and, Nathalie Estephan: nathalieestephan@usek.edu.lb).

## Supplementary Materials

The following supporting information can be downloaded at: https://www.mdpi.com/article/10.3390/plants12142681/s1; Table S1: Comparison of total phenols data (mg GAE/Kg of oil) obtained in the present study with data from previous works [32,33,34,64]; Table S2, Comparison of C18:1 percentages (%) obtained in the present study with data from previous studies [26,28,30,34,40,41,42,43,53,64,65,66,67,68,69].; Table S3, Comparison of the contents (mg/kg) of the secoiridoid derivatives oleacein (3,4-DHPEA-EDA) and oleocanthal (p-HPEA-EDA) obtained in the present study with those obtained in previous studies [26,41,50,51,52,53,70].

## References

[1] Guerrero N., López M., Caudullo G., de Rigo D., San-Miguel-Ayanz J., de Rigo D., Caudullo G., Houston Durrant T., Mauri A. (2016). Olea Europaea in Europe: Distribution, Habitat, Usage and Threats.

[2] Angerosa F., Mostallino R., Basti C., Vito R. (2000). Virgin olive oil odour notes: Their relationships with volatile compounds from the lipoxygenase pathway and secoiridoid compounds. Food Chem..

[3] Inglese P., Famiani F., Galvano F., Servili M., Esposto S., Urbani S. (2011). Factors Affecting Extra-Virgin Olive Oil Composition. Horticultural Reviews.

[4] El Riachy M., Priego-Capote F., León L., Rallo L., Castro M. (2011). Hydrophilic antioxidants of virgin olive oil. Part 1: Hydrophilic phenols: A key factor for virgin olive oil quality. Eur. J. Lipid Sci. Technol..

[5] Jimenez-Lopez C., Carpena M., Lourenço-Lopes C., Gallardo-Gomez M., Lorenzo J.M., Barba F.J., Prieto M.A., Simal-Gandara J. (2020). Bioactive Compounds and Quality of Extra Virgin Olive Oil. Foods.

[6] Lombardo L., Grasso F., Lanciano F., Loria S., Monetti E. (2018). Broad-Spectrum Health Protection of Extra Virgin Olive Oil Compounds. Stud. Nat. Prod. Chem..

[7] Mariotti M., Peri C., Peri C. (2014). The composition and nutritional properties of extra-virgin olive oil. The Extra-Virgin Olive Oil Handbook.

[8] Sánchez-Villegas A., Sánchez-Tainta A. (2018). Chapter 4—Virgin Olive Oil: A Mediterranean Diet Essential. The Prevention of Cardiovascular Disease through the Mediterranean Diet.

[9] Gorzynik-Debicka M., Przychodzen P., Cappello F., Kuban-Jankowska A., Marino Gammazza A., Knap N., Wozniak M., Gorska-Ponikowska M. (2018). Potential Health Benefits of Olive Oil and Plant Polyphenols. Int. J. Mol. Sci..

[10] Saenz M.T., Garcia M.D., Ahumada M.C., Ruiz V. (1998). Cytostatic activity of some compounds from the unsaponifiable fraction obtained from virgin olive oil. Farmaco.

[11] Tsimidou M., Blekas G.D. (2002). Olive oil. Encyclopedia of Food Sciences and Nutrition.

[12] Ceci L., Carelli A. (2010). Relation Between Oxidative Stability and Composition in Argentinian Olive Oils. J. Am. Oil Chem. Soc..

[13] Kalua C.M., Allen M.S., Bedgood D.R., Bishop A.G., Prenzler P.D. (2005). Discrimination of olive oils and fruits into cultivars and maturity stages based on phenolic and volatile compounds. J. Agric. Food Chem..

[14] Mele M., Islam M., Kang H.M., Giuffrè A. (2018). Pre-and post-harvest factors and their impact on oil composition and quality of olive fruit. Emir. J. Food Agric..

[15] El Qarnifa S., El Antari A., Hafidi A. (2019). Effect of Maturity and Environmental Conditions on Chemical Composition of Olive Oils of Introduced Cultivars in Morocco. J. Food Qual..

[16] Ollivier D., Boubault E., Pinatel C., Souillol S., Guérère M., Artaud J. (2004). Analyse de la fraction phenolique des huiles d’olive vierges. Food Chem. Toxicol..

[17] El Riachy M., Priego-Capote F., Rallo L., Castro M., León L. (2012). Phenolic profile of virgin olive oil from advanced breeding selections. Span. J. Agric. Res..

[18] Calò F., Girelli C.R., Wang S.C., Fanizzi F.P. (2022). Geographical Origin Assessment of Extra Virgin Olive Oil via NMR and MS Combined with Chemometrics as Analytical Approaches. Foods.

[19] Maestrello V., Solovyev P., Bontempo L., Mannina L., Camin F. (2022). Nuclear magnetic resonance spectroscopy in extra virgin olive oil authentication. Compr. Rev. Food Sci. Food Saf..

[20] Valli E., Bendini A., Berardinelli A., Ragni L., Riccò B., Grossi M., Gallina Toschi T. (2016). Rapid and innovative instrumental approaches for quality and authenticity of olive oils. Eur. J. Lipid Sci. Technol..

[21] Armenta S., Moros J., Garrigues S., Guardia M.D.L. (2010). The Use of Near-Infrared Spectrometry in the Olive Oil Industry. Crit. Rev. Food Sci. Nutr..

[22] Shaik R. (2008). Analytical Tool for Rapid Analysis of Edible Oil. Master’s Thesis.

[23] Cayuela J., García J., Caliani N. (2009). NIR prediction of fruit moisture, free acidity and oil content in intact olives. Grasas Y Aceites.

[24] Stat F. https://www.fao.org/faostat/en/#data/QCL.

[25] IDAL. http://investinlebanon.gov.lb/Content/uploads/SideBlock/171011013554317~{}Olive%20Oil%20Factsheet%202017.pdf.

[26] El Riachy M., Bou-Mitri C., Youssef A., Andary R., Skaff W. (2018). Chemical and Sensorial Characteristics of Olive Oil Produced from the Lebanese Olive Variety ‘Baladi’. Sustainability.

[27] Gharbi I., Issaoui M., Mehri S., Cheraief I., Sifi S., Hammami M. (2015). Agronomic and Technological Factors Affecting Tunisian Olive Oil Quality. Agric. Sci..

[28] Rondanini D., Castro D., Searles P., Rousseaux M. (2011). Fatty acid profiles of varietal virgin olive oils (*Olea europaea* L.) from mature orchards in warm arid valleys of Northwestern Argentina (La Rioja). Grasas Y Aceites.

[29] Rondanini D.P., Castro D.N., Searles P.S., Rousseaux M.C. (2014). Contrasting patterns of fatty acid composition and oil accumulation during fruit growth in several olive varieties and locations in a non-Mediterranean region. Eur. J. Agron..

[30] El Riachy M., Hamade A., Ayoub R., Dandachi F., Chalak L. (2019). Oil Content, Fatty Acid and Phenolic Profiles of Some Olive Varieties Growing in Lebanon. Front. Nutr..

[31] Villa P. (2003). La Culture de L’olivier.

[32] Ripa V., Rose F., Caravita M.A., Parise M.R., Perri E., Rosati A., Pandolfi S., Paoletti A., Pannelli G., Padula G. (2008). Qualitative evaluation of olive oils from new olive selections and effects of genotype and environment on oil quality. Adv. Hortic. Sci..

[33] Tura D., Gigliotti C., Pedò S., Failla O., Bassi D., Serraiocco A. (2007). Influence of cultivar and site of cultivation on levels of lipophilic and hydrophilic antioxidants in virgin olive oils (*Olea europea* L.) and correlations with oxidative stability. Sci. Hortic..

[34] Rotondi A., Alfei B., Magli M., Pannelli G. (2010). Influence of genetic matrix and crop year on chemical and sensory profiles of Italian monovarietal extra-virgin olive oils. J. Sci. Food Agric..

[35] Piravi-Vanak Z., Ghasemi J.B., Ghavami M., Ezzatpanah H., Zolfonoun E. (2012). The Influence of Growing Region on Fatty Acids and Sterol Composition of Iranian Olive Oils by Unsupervised Clustering Methods. J. Am. Oil Chem. Soc..

[36] Ollivier D., Artaud J., Pinatel C., Durbec J.P., Guérère M. (2003). Triacylglycerol and fatty acid compositions of French virgin olive oils. Characterization by chemometrics. J. Agric. Food Chem..

[37] Aparicio M., Cano N., Chauveau P., Azar R., Canaud B., Flory A., Laville M., Leverve X. (1999). Nutritional status of haemodialysis patients: A French national cooperative study. French Study Group for Nutrition in Dialysis. Nephrol. Dial. Transplant..

[38] Frankel E.N. (2011). Nutritional and biological properties of extra virgin olive oil. J. Agric. Food Chem..

[39] Beltrán G., del Río C., Sánchez S., Martínez L. (2004). Seasonal changes in olive fruit characteristics and oil accumulation during ripening process. J. Sci. Food Agric..

[40] Mailer R., Ayton J., Graham K. (2010). The Influence of Growing Region, Cultivar and Harvest Timing on the Diversity of Australian Olive Oil. J. Am. Oil Chem. Soc..

[41] Dabbou S., Dabbou S., Chehab H., Taticchi A., Servili M., Hammami M. (2015). Content of Fatty Acids and Phenolics in Coratina Olive Oil from Tunisia: Influence of Irrigation and Ripening. Chem. Biodivers..

[42] Aguilera M.P., Beltrán G., Ortega D., Fernández A., Jiménez A., Uceda M. (2005). Characterisation of virgin olive oil of Italian olive cultivars: ‘Frantoio’ and ‘Leccino’, grown in Andalusia. Food Chem..

[43] Chehade A., Bitar A., Aline K., Choueiri E., Nabbout R., Youssef C., Smeha D., Awada C., Al Chami Z., Dubla E. (2012). Characterization of the Main Lebanese Olive Germplasm.

[44] García-González D.L., Aparicio-Ruiz R., Aparicio R. (2008). Virgin olive oil-Chemical implications on quality and health. Eur. J. Lipid Sci. Technol..

[45] El-Gharbi S., Tekaya M., Bendini A., Valli E., Palagano R., Hammami M., Gallina Toschi T., Mechri B. (2018). Effects of Geographical Location on Chemical Properties of Zarazi Virgin Olive Oil Produced in the South of Tunisia. Am. J. Food Technol..

[46] Manai-Djebali H., Krichène D., Ouni Y., Gallardo L., Sánchez J., Osorio E., Daoud D., Guido F., Zarrouk M. (2012). Chemical profiles of five minor olive oil varieties grown in central Tunisia. J. Food Compos. Anal..

[47] Mansour A.B., Gargouri B., Flamini G., Bouaziz M. (2015). Effect of agricultural sites on differentiation between Chemlali and Neb Jmel olive oils. J. Oleo Sci..

[48] Cicerale S., Conlan X.A., Sinclair A.J., Keast R.S. (2009). Chemistry and health of olive oil phenolics. Crit. Rev. Food Sci. Nutr..

[49] Cicerale S., Lucas L.J., Keast R.S. (2012). Antimicrobial, antioxidant and anti-inflammatory phenolic activities in extra virgin olive oil. Curr. Opin. Biotechnol..

[50] Ragusa A., Centonze C., Grasso M.E., Latronico M.F., Mastrangelo P.F., Fanizzi F.P., Maffia M. (2017). Composition and Statistical Analysis of Biophenols in Apulian Italian EVOOs. Foods.

[51] Gonzalez D., Romero N., Aparicio R. (2010). Comparative Study of Virgin Olive Oil Quality from Single Varieties Cultivated in Chile and Spain. J. Agric. Food Chem..

[52] Servili M., Esposto S., Veneziani G., Urbani S., Taticchi A., Di Maio I., Selvaggini R., Sordini B., Montedoro G. (2011). Improvement of bioactive phenol content in virgin olive oil with an olive-vegetation water concentrate produced by membrane treatment. Food Chem..

[53] Ben-Hassine K., Taamalli A., Rezig L., Yangui I., Benincasa C., Malouche D., Kamoun N., Mnif W. (2022). Effect of processing technology on chemical, sensory, and consumers’ hedonic rating of seven olive oil varieties. Food Sci. Nutr..

[54] Uceda M., Hermoso M., Barranco D., Fernández-Escobar R., Rallo L. (1998). El Cultivo del Olivo, AAVV.

[55] Martínez-Suarez J.M., Alba J., Lanzón A. (1975). Informe sobre la utilización del Analizador de Rendimientos “Abencor”. Grasas Y Aceites.

[56] Union R.o.t.E. https://eur-lex.europa.eu/legal-content/EN/TXT/PDF/?uri=CELEX:01991R2568-20161204&from=IT.

[57] Montedoro G.F., Servili M., Baldioli M., Miniati E. (1992). Simple and hydrolyzable phenolic compounds in virgin olive oil. 1. Their extraction, separation, and quantitative and semiquantitative evaluation by HPLC. J. Agric. Food Chem..

[58] Singleton V.L., Rossi J.A. (1965). Colorimetry of total phenolics with phosphomolybdic -phosphotungstic acid reagents. Am. J. Enol. Vitic..

[59] Bertrand D., Dufour E. (2006). La Spectroscopie Infrarouge et ses Applications Analytiques.

[60] Guo Q., Wu W., Massart D.L. (1999). The robust normal variate transform for pattern recognition with near-infrared data. Anal. Chim. Acta.

[61] Bouveresse D.J.R., Rutledge D.N. (2012). Introduction à l’analyse en composantes indépendantes et comparaison avec l’analyse en composantes principales. Ann. Falsif. L’expertise Chim. Toxicol..

[62] Cowe I.A., McNicol J.W. (1985). The Use of Principal Components in the Analysis of Near-Infrared Spectra. Appl. Spectrosc..

[63] Huberty C.J. (1994). Applied Discriminant Analysis.

[64] Škevin D., Rade D., Štrucelj D., Mokrovšak Ž., Neđeral S., Benčić Đ. (2003). The influence of variety and harvest time on the bitterness and phenolic compounds of olive oil. Eur. J. Lipid Sci. Technol..

[65] Carelli A. (2008). Olive Oil Chemistry in Argentina.

[66] Zarrouk W., Bechir B., Taamalli W., Daoud D., Zarrouk M. (2009). Oil fatty acid composition of eighteen Mediterranean olive varieties cultivated under the arid conditions of Boughrara (southern Tunisia). Grasas Y Aceites.

[67] Poiana M., Mincione A. (2004). Fatty acids evolution and composition of olive oils extracted from different olive cultivars grown in Calabrian area. Grasas Y Aceites.

[68] León L., de la Rosa R., Gracia Torres A., Diego B., Rallo L. (2008). Fatty acid composition of advanced olive selections obtained by crossbreeding. J. Sci. Food Agric..

[69] Pinelli P., Galardi C., Mulinacci N., Vincieri F.F., Cimato A., Romani A. (2003). Minor polar compound and fatty acid analyses in monocultivar virgin olive oils from Tuscany. Food Chem..

[70] Gambacorta G., Faccia M., Previtali M.A., Pati S., Notte E., Baiano A. (2010). Effects of Olive Maturation and Stoning on Quality Indices and Antioxidant Content of Extra Virgin Oils (cv. Coratina) during Storage. J. Food Sci..

