# Supplementary material for: Fatty Acid and Phenolic Profiles of Virgin Olive Oils from Local and European Varieties Planted in Lebanon
Source: Plants (Basel). 2023 Jul 18;12(14):2681. doi: 10.3390/plants12142681 (PMC10386562; doi:10.3390/plants12142681)
Supplement: Supplementary file 1 [file plants-12-02681-s001.zip › plants-2408092-supplementary.pdf]

Supplementary Materials

Fatty Acid and Phenolic Profiles of Virgin Olive Oils from Local and European Varieties Planted in Lebanon

Milad El Riachy [1,*], Peter Moubarak [1], Ghenwa Al Hawi [1], Myriam Geha [1], Walid Mushantaf [2], Nathalie Estephan [3,*] and Wadih Skaff [4]

1 Department of Olive and Olive Oil, Lebanese Agricultural Research Institute, Zahleh P.O. Box 287, Lebanon
2 Boustan Al Zaytoun, Aabra P.O. Box 175-646, Lebanon
3 Department of Chemistry and Biochemistry, Faculty of Arts and Sciences, Holy Spirit University of Kaslik, Jounieh P.O. Box 446, Lebanon
4 Food Industry and Agriculture Unit, ESIAM, Faculty of Engineering, Saint Joseph University of Beirut, Beirut P.O. Box 17-5208, Lebanon
* Correspondence: mraichy@lari.gov.lb (M.E.R.); nathalieestephan@usek.edu.lb (N.E.);
Tel.: +961-3243711 (M.E.R.); +961-9600933 (N.E.)

Table S1. Comparison of total phenols data (mg GAE/Kg of oil) obtained in the present study with data from previous works [32–34,64].

	**Present study**	**Ripa et al., (2008)**[32]		**Škevin et al. (2003)** [64]			**Tura et al. (2007)** [33]			**Rotondi et al. (2010)** [34]
		Geographical origin		Harvest			Mixture 1	Mixture 2	Mixture 3	
		Metaponto	Rossano	1	2	3				
**Bianchera**	586.64 ± 220.16			382±80	338±116	305±50				-
**Coratina**	574.06 ± 193.67	157±35	147±2.7							500 - 550
**Maurino**	446.64 ± 101.15						181.2			
**Pendolino**	433.34 ± 159.92						150.5		181.5	
**Leccino**	292.53 ± 182.04	19±13.8	81±36	277±81	175±49	130±33	160.9	247.6	150.6	350 - 400
**Frantoio**	295.01 ± 73.04						175.7	251.9	175.5	450 - 500

Table S2. Comparison of C18:1 percentages (%) obtained in the present study with data from previous studies [26,28,30,34,40–43,53,64–69].

**Variety**	**Present study**	**Dabbou et al. (2015) [41]**	**Rondanini et al. (2011) [28]**	**Carelli (2008) [65]**	**Zarrouk et al. (2009) [66]**	**Ben Hassine et al. (2022) [53]**	**Mailer et al. (2010) [40]**	**Rotondi et al. (2010) [34]**	**Poiana and Mincione (2004) [67]**	**Škevin et al. (2003) [64]**	**Aguilera et al. (2005) [42]**	**León et al. (2008) [68]**	**Pinelli et al. (2003) [69]**	**El Riachy et al. (2019) [30]**	**Chehade et al. (2012) [43]**	**El Riachy et al. (2018) [26]**
**Coratina**	73.88	72.10–75.83	68.9	64.6–69.4	70.88	75.35–79.1	74.0–81.8	78.37	78.10							
**Bianchera**	71.12									75.31–75.97						
Pendolino	71.54								75.48							
Frantoio	70.54		63.1				65.7–80.3	76.9			70.9–78.3	67.09	75.2–75.7			
Leccino	70.13		67.5		67.9		73.1–77.9	75.56	73.45	74.38–75.69	74.1–77.8					
**Baladi**	67.46													67.09–67.63	62.44–65.95	69.42–71.46
**Soury**	66.65														67.28–67.55	

Table S3. Comparison of the contents (mg/kg) of the secoiridoid derivatives oleacein (3,4-DHPEA-EDA) and oleocanthal (p-HPEA-EDA) obtained in the present study with those obtained in previous studies [26,41,50–53,70].

Variety	Present study		**Servili et al. (2011) [52]**		**Dabbou et al. (2015) [41]**		**Gambacorta et al. (2010) [70]**		**Ben Hassine et al. (2022) [53]**		**Ragusa et al. (2017) [50]**		**González et al. (2010) [51]**		**El Riachy et al. (2018) [26]**	
	Oleacein	Oleocanthal	Oleacein	Oleocanthal	Oleacein	Oleocanthal	Oleacein	Oleocanthal	Oleacein	Oleocanthal	Oleacein	Oleocanthal	Oleacein	Oleocanthal	Oleacein	Oleocanthal
Coratina	109.96	70.26	282	216	144.83	106.65	29.72–32.21	31.98–55.61	10.77	20.86						
Frantoio	12.52	21.16									12.5	20.1	38.41	41.41		
Leccino	140.27	120.03									44	14.8	38.93	10.91		
Baladi	16.24	26.36													26.97–27.78	48.36 – 73.53
Soury	25.05	73.02														
